# The Relationship between Body Composition and Physical Activity Level in Students of Medical Faculties

**DOI:** 10.3390/jcm13010050

**Published:** 2023-12-21

**Authors:** Aleksandra Jaremków, Iwona Markiewicz-Górka, Wojciech Hajdusianek, Karolina Czerwińska, Paweł Gać

**Affiliations:** Department of Population Health, Division of Environmental Health and Occupational Medicine, Wroclaw Medical University, Mikulicza-Radeckiego 7, 50-368 Wrocław, Poland

**Keywords:** accelerometry, body composition, health, physical activity, students

## Abstract

Maintaining an active lifestyle is crucial for good health. Markers of health risk include tissue components. This study aimed to indicate which body composition elements create the strongest correlations with physical activity performed in young students of medical faculties. The study group consisted of 75 students (33 men and 42 women) from Wroclaw Medical University. Each student underwent accelerometer and anthropometric measurements and body composition analysis. Both men and women had similar physical activity levels. The study found that the amount of vigorous physical activity correlated significantly with the basal metabolic rate (BMR), fat, water and muscle content, fat-free mass (FFM), bone mass, extracellular to intracellular water ratio (ECW/ICW), and phase angle (PA), with r~ ± (0.2–0.4). The amount of moderate physical activity correlated with body mass, body mass index (BMI), BMR, FFM, and bone mass, with r~0.3–0.5. There are dimorphic differences in the strength of correlations between physical activity and elements of body composition. A greater amount of moderate and vigorous physical activity is associated with greater FFM and bone mass in men, which causes BMI to increase as well (in this case, higher BMI is not a sign of being overweight). For women, the beneficial effect of higher amounts of vigorous physical activity on reducing fat content and increasing muscle mass is more pronounced. In both men and women, an improvement in hydration is evident with increased vigorous physical activity volume.

## 1. Introduction

The risk of developing lifestyle diseases, such as diabetes, obesity or cardiovascular disease, is decreased by a healthy lifestyle and physical activity in particular [1,2,3]. World Health Organization (WHO) recommendations include general, as well as age/pregnancy/disease/disability-adopted actions recommended for maintaining good health and physical condition.

In general, adults should do at least 150–300 min/week of moderate or 75–150 min/week of vigorous exercise [4]. Activities considered moderate include, for example, walking very brisk (4 mph), heavy cleaning (washing windows, vacuuming, mopping), mowing lawn (power mower), bicycling light effort (10–12 mph), recreational badminton and tennis doubles. Vigorous activities include, for example, hiking, jogging at 6 mph, shoveling, carrying heavy loads, fast bicycling (14–16 mph), basketball game, soccer game and tennis singles. One of the ways to assess physical activity level is the moderate-to-vigorous physical activity (MVPA). MVPA duration is the sum of moderate and vigorous physical activity (time in minutes) that adults do in a week [5].

The mounting evidence that regular physical activity benefits health led to the development of modern and more precise methods to measure its level. One of the most used instruments is an accelerometer. This device measures acceleration in three axes (x, y, z). It can be placed in various parts of a human body, most commonly wristband, waist, or ankle. Modern apparatus (e.g., GENEActiv) consists of memory of up to 0.5 GB and can collect data with up to 100 Hz frequency. The device has two main advantages: its small size and a long-lasting battery that can last up to 2 months. It also has temperature sensors that ensure the accelerometer is not removed during measurement. According to research, the GENActiv is highly accurate with a VO_2_ criterion validity of r = 0.86 and 0.83 for the nondominant and dominant wrist, respectively. Due to the obtained results of validity and the designated cut-off points for types of physical activity, from sedentary to vigorous, accelerometers are considered reliable devices to measure physical activity levels [6]. Nevertheless, many studies still rely on survey data collection [7,8,9], which may not be as accurate as mechanical/electronic measuring devices, which is confirmed by numerous comparative studies [10,11,12].

Body composition characteristics (e.g., content of water, fat, muscles, and bones in the body) are most often determined by the noninvasive BIA (bioelectrical impedance analysis) method using the so-called body composition analyzer. It is a device whose task is to assess the distribution of extra- and intracellular fluids in the body [13]. Electrical resistance, i.e., resistance (R) and reactance (Xc), is measured. It consists in applying a low-intensity (0.8–1 mA) and high-frequency electric current (approx. 50 kHz) to the skin [14]. The patient stands with his bare feet on the scale platform, where the electrodes are placed, and holds two holders, also with electrodes, in his hands [15]. During this time, current flows through the body through electrolyte solutions, while resistance is measured through adipose tissue/extracellular water. Cell membranes act as a capacitor. Two covers of hydrophilic phospholipids conduct electricity, while the dielectric layer, which is formed by lipophilic fragments—on the contrary. This allows electrical charges to build up on each side of the membrane, which allows the reactance to be measured. This causes a phase shift of the applied electric current, which is referred to as the phase angle [14,15]. 

So far, the analysis of links between body composition elements and physical activity has only been partially conducted, considering only selected, most popular indicators: body fat content, body mass index (BMI), and waist circumference [10,11]. These are obesity indicators, the decrease of which was noted with increased physical exercise. However, there are no clear answers to the question about the relationship between physical activity intensity and body composition. The conducted research, which partly touches on the analyzed problem, is, e.g., the article of Dewi et al. [16]. The authors found that adipose tissue content is a more accurate indicator of low physical fitness in obese individuals than BMI (stronger correlation between body fat content and physical fitness than between BMI and physical fitness). A group of scientists from Latin America [17], apart from positive (weak) correlations between a sedentary lifestyle and BMI and waist circumference, also showed a correlation with the respondents’ neck circumference. Thus, more studies that examine a wider range of factors related to physical activity and body composition are required.

This study aimed to indicate which body composition elements create the strongest correlations with physical activity performed in young students of medical faculties. The obtained result may influence the improvement/maintenance of their proper body composition and health condition, and thus their quality of life.

## 2. Materials and Methods

Group size was determined using a sample size calculator. The selection conditions were as follows: population size 150,000 (number of students in the academic center), fraction size 0.1 (percentage of students in medical faculties), maximum error 10%, confidence level 95%. The minimum sample size was estimated at 35.

The study group comprised 75 students from Wroclaw Medical University, mainly from the Faculty of Medicine, but also from the Faculty of Pharmacy and The Faculty of Health Sciences. Most of the participants were in their first or second year of studies. To qualify for the study, individuals had to be healthy and not suffer from chronic illnesses, such as cardiovascular diseases or type 2 diabetes, that could affect their body composition elements. 

The study group consisted of both men and women, with women making up 56% and men 44%. The average age of participants was 21 years. Out of the 75 students, six had completed higher education studies before. For the remaining respondents, medical studies were the first studies. More than half of the participants were single, and among those who had a partner, only one was married. During their studies, only four participants had gainful employment. Most of the participants lived in the academic city, with the majority residing in blocks of flats, while fewer lived in single-family or tenement houses. Further information about the studied group is presented in Table 1.

This study was approved by the Bioethics Committee of Wroclaw Medical University in Poland on 29 January 2021 (KB-19/2021).

The study involved questionnaire testing, accelerometer testing, and the measurement of anthropometric and body composition characteristics. All participants were fully informed about the study’s goals and procedures and provided their voluntary consent.

During the study, the participants wore a three-axis accelerometer GENEActiv on their nondominant wrist for seven days straight. This device recorded their daily activities and measured their physical activity. Its reliability and accuracy were described in a study by Esliger et al. [6]. The participants were instructed to wear the device at all times, including during sleep and bathing, as it is waterproof. The frequency of data acquisition was 100 Hz. The week that the device was worn corresponded with the week participants referred to in the questionnaire. The data were then collected from the device in 60 s time intervals (epochs) with a resolute GENEActiv software (https://apps.microsoft.com/detail/9NTLZLBXNHR6?hl=en-US&gl=US, accessed on 24 September 20). The following equation was used:

SVM_gs_ = Σ[(x^2^ + y^2^ + z^2^)^1/2^ − 1g]

SVM_gs_—sum of vector magnitude in three dimensions with gravity subtracted g—9.81 m/s^2^ (gravitation unit).

Time spent sleeping was deducted, and the collected data were grouped based on specific cut-off points [6]. Then, the amount of time spent on different types of physical activity for each participant was calculated, and the results as MET-min/week in relevant physical activity categories were presented [18]:

1. Sedentary (<1.5 MET)
2. Light (1.5–2.99 MET)
3. Moderate (3.0–5.99 MET)
4. Vigorous (≥6 MET).

The TANITA MC-780 MA analyzer was used to perform body composition analysis. This involved using the bioelectrical impedance method. The following characteristics were considered: height, body mass, body mass index (BMI), hip circumference (HC), waist circumference (WC), basal metabolic rate (BMR), fat, water and muscle percentage, fat-free mass (FFM), bone mass, extracellular water/intracellular water ratio (ECW/ICW ratio) and phase angle value. To ensure accuracy, the official manufacturer guidelines for taking measurements were followed, which required participants to be barefoot, wearing light clothing, without any jewelry, and with empty pockets.

It was also ensured that the participants met the following conditions: at least 3 h after waking up, at least 3 h after the last meal, at least 12 h after heavy physical activity, at least 12 h after alcohol intake, and urinating before the measurement [19].

Participants who were female and currently menstruating, pregnant, or had medical implants or other transmitting electrical signal devices were excluded from the study [20]. As a result, a total of 2 participants were excluded.

A stadiometer was used to obtain participants’ height (accurate to 0.1 cm). Centimeter tapes were used to obtain waist and hip circumferences (also accurate to 0.1 cm). Each measurement was taken twice and averaged for accuracy. The waist-to-hip ratio (WHR) was measured in a group of overweight participants according to WHO guidelines [21] (android obesity WHR > 0.9 in males and ≥0.85 in females; gynoid obesity WHR < 0.9 in males and <0.85 in females).

Statistica 13 software for Windows was used to perform statistical analysis. The quantitative data were tested for normal distribution (Shapiro–Wilk normality test). On the part of the data, a logarithmic transformation of variables was performed to obtain a normal distribution. To compare physical activity between males and females, the Student’s t-test was used. To investigate the relationship between the amount of time spent on the activity of a given type (light, moderate, vigorous) and somatic features (from anthropometry and BIA), Pearson correlations were used. The multiple regression analysis (stepwise method) was also performed to test the influence of different intensities of physical activity on the anthropometric and body composition elements. For all statistical calculations, a significance level of *p* < 0.05 was used.

## 3. Results

Table 2 displays the anthropometric and body composition characteristics of the study group. Of all participants, 80% had a normal BMI. However, 12% were overweight, with 4% of females and 8% of males experiencing gynoid obesity.

As part of the analysis, participants’ physical activity was assessed by calculating the level of MVPA. The study group had an average MVPA of 851.1 min/week. 

There were no significant differences in physical activity between males and females, as shown in Table 3.

The male group showed the strongest correlations between physical activity and anthropometric and body composition elements (Table 4). In terms of the physical effort involved, the strongest correlations were found between the amount of time of vigorous physical activity and various body composition characteristics, such as BMR, FFM, bone mass (in males), PA (in males and all participants), ECW/ICW ratio (in males, females, and all participants), fat, water, and muscle content (in females and all participants). Within the male group, slightly fewer correlations were found between moderate physical activity and body composition elements such as body mass, BMR, FFM, bone mass, and BMI. For light physical activity, only males showed a correlation between bone mass and ECW/ICW ratio. Total MET was linked to BMI and BMR in males, while in females and all participants, it was associated with P.

Based on multivariate analysis, it was found that only moderate and vigorous physical activity had a significant impact on the tested elements. For males, moderate physical activity was associated with body mass, BMI, and BMR, while vigorous activity was significantly correlated with an increase in muscle percentage for females, as indicated in Table 5. 

Furthermore, Table 6 shows that vigorous activity was linked to fat, water, muscle content, ECW/ICW ratio, and phase angle value for all participants.

## 4. Discussion

Our study confirms the existence of many different relationships between body composition elements and physical activity of students of medical faculties. Few studies of this type have been conducted on such a research group. Previous research [22,23] mainly focused on the relationship between body composition and physical fitness (e.g., sit and reach, run, sprint, etc.) rather than different types of physical activity (sedentary, light, moderate, vigorous physical activity). This is even more interesting because these are students of medical faculties, aware of the importance of physical exercise for the health of the body, so the relationships obtained in this group should be exemplary for all young people. Their correct approach to physical exercise is proven by the MVPA index they achieved, which significantly exceeded the WHO guidelines.

In this study, body composition characteristics correlated mostly with the amount of vigorous and moderate physical activity. That suggests the high importance of such physical effort on the overall condition of the human body. It is important to note that through vigorous exercise, the body burns fat and increases muscle and water percentage, leading to a decrease in ECW/ICW ratio and an increase in phase angle [15], as also proven by the presented studies. As muscles are mostly comprised of water (78%), an increase in muscle mass leads to an increase in the water ratio and thus reduces the fat ratio in the body [24]. The ECW/ICW ratio is closely tied to the phase angle value (in a reverse relation). Demonstrated ECW/ICW ratio indicates cell hydration and wall integrity, which affects many of the body’s processes and can express muscle strength [25]. The phase angle value indicates the electric potential of cell membranes and their nutrition, as well as their integrity and permeability, which is linked to the ECW/ICW ratio. The value of the phase angle is an indicator of the cell membrane’s electric potential and a marker of cells’ nutrition. It also gives information about cell wall integrity and permeability and, therefore, is connected to the ECW/ICW ratio. In healthy individuals, the phase angle value should be around five to seven degrees. A decrease below five degrees may indicate issues with cell wall function, potentially caused by neoplasms, malnutrition, or physical exhaustion [26].

Both vigorous and moderate physical activity increases BMR, which has already been proven [27]. This is likely due to an increase in fat-free mass, particularly bones and muscles [28]. Having greater muscle mass makes the body more efficient at using energy, which leads to a higher metabolism. Interestingly, moderate physical activity caused an increase in body mass, consequently, BMI. However, this increase was not caused by an increase in fat tissue but rather by an increase in lean tissue. These were the strongest correlations obtained in this study. Therefore, it can be assumed that BMR, BMI, FFM and bone mass are the most sensitive elements to these types of physical activity. This allows us to assume that among the various body composition elements, they are the most important in the process of improving/maintaining the correct body composition and the health condition of the body.

Increasing time spent on light physical activity led to a decrease in bone mass and an unfavorable increase in the ECW/ICW ratio. This indicates that light physical activity is inefficient and does not lead to improvement of body composition elements. Total MET, considering all types of physical activity performed during the week, contributed to an increase in BMI, BMR and phase angle. A positive correlation between those elements was obtained for moderate and vigorous physical activity.

It is worth noting that our study is the first to analyze this number of body composition elements in relation to physical activity. To our knowledge, previously, only a few indicators were correlated with physical activity—most commonly, fat content, BMI, body mass, and waist circumference, which does not fully explain the changes in body composition in people who exercise. In most cases, previous studies stay consistent with our results. However, there are some differences. In studies by Guo et al. [10] and Sabia et al. [11] a decrease in obesity characteristics, such as BMI, adipose tissue content, and waist circumference, correlated with increased physical activity. However, contrary to our study, their research had a higher number of obese participants, and, therefore, the decrease in BMI was mainly caused by a decrease in adipose tissue content. It is not known how other elements developed, i.e., FFM, muscle mass, water content, ECW/ICW, and phase angle, which can also be crucial in relation to different types of physical activity. Such information could help guide exercise selection of a certain intensity to rationally/healthy maintain a proper figure and improve the health condition of the body. They can also enable a better understanding of the mechanisms taking place in our body based on the network of connections between various body composition elements and physical activity. 

A study by Savikangas et al. [29] highlighted the significance of physical activity in burning fat. Unlike our findings, the study noted that even light physical activity had a positive impact. However, the difference in the age and lifestyle of the study groups may have influenced this result. The participants in their study were older, sedentary, and obese compared to our study group. Therefore, even a slight increase in physical activity could have led to a reduction in body fat. However, not much is known about the other elements of body composition and which of them also undergo changes under the influence of this type of physical activity. Such information could deepen the knowledge about the mechanisms of weight loss in older, obese people, as well as result in a new exercise program. That is why it is so important to know the full view of changes in body composition elements that occur during physical exercise.

It is important to note that individuals may engage in multiple types of physical activity during the analyzed period. The multivariate analysis allows for the consideration of all activities at once. This approach helps identify which activities are most influential in affecting body composition elements, which are less significant, and which have no impact at all. After performing the multivariate analysis, a significantly lower number of relations between physical activity levels and selected body composition elements were found than in a Pearson correlation analysis. The multivariate analysis showed that engaging in moderate to vigorous exercise is crucial for maintaining a healthy body and proper body composition elements. For men, engaging in moderate exercise may increase body weight and BMI, but it also leads to an increase in lean mass and muscle mass, resulting in a higher BMR. In females, only vigorous physical activity increased the percentage of muscles. In both groups, vigorous activity resulted in adipose tissue burning, an increase in water and muscle content, a decrease in ECW/ICW, and an increase in the phase angle.

Our study is limited by a relatively small group of participants within a specific age range (students of medical faculties—mainly the first and second year of studies), which makes it challenging to apply the obtained results to the entire population. This limitation was caused by the COVID-19 pandemic, which hindered our ability to recruit more participants. The exclusion of two students from the study based on the adopted exclusion criteria for body composition analysis should be considered a limitation of the study. Exclusion criteria for body composition analysis adopted according to the manufacturer’s instructions for the research device are questionable. 

## 5. Conclusions

Among the analyzed elements of body composition, physical activity, especially vigorous and moderate physical exercise, had the greatest impact on BMI, BMR, FFM and bone mass (the strongest correlations). This proves that they are most sensitive to these types of physical activity. It can, therefore, be assumed that these elements will be of decisive importance in the process of improving/maintaining proper body composition and health condition. There are dimorphic differences in the strength of correlations between physical activity and elements of body composition. A greater amount of moderate and vigorous physical activity is associated with greater FFM and bone mass in men. For women, the beneficial effect of higher amounts of vigorous physical activity on reducing fat content and increasing muscle mass is more pronounced. In both men and women, an improvement in hydration is evident with increased vigorous physical activity volume.

## Figures and Tables

**Table 1:** Characteristics of the participants; n = 75.

	Mean ± SD or n (%)
Age (years, Mean ± SD)	20.9 ± 1.5
Sex (n (%))	
Female	42 (56.0)
Male	33 (44.0)
Education (n (%))	
Secondary	69 (92.0)
Higher	6 (8.0)
Marital status (n (%))	
Single	41 (54.7)
Partner in a relationship	33 (44.0)
Married	1 (1.3)
Employment status (n (%))	
Unemployed	71 (94.7)
Employed	4 (5.3)
Current place of residence (n (%))	
A very large city: >100,000 residents	61 (81.3)
Large city: 50,000–100,000 residents	4 (5.3)
City: 10,000–50,000 residents	4 (5.3)
A small town: 10,000 residents	2 (2.7)
Village	4 (5.3)
Accommodation type (n (%))	
Block of flats	48 (64.0)
Detached house	16 (21.3)
Multi-family house/tenement house	11 (14.7)

**Table 2:** Anthropometric and body composition characteristics of the studied population.

Characteristics	Total [Mean ± SD]	Men [Mean ± SD]	Women [Mean ± SD]
Height (cm)	173.7 ± 9.4	181.3 ± 7.0	167.8 ± 6.1
Body mass (kg)	65.8 ± 12.1	74.3 ± 10.2	59.1 ± 9.0
BMI (kg/m^2^)	21.7 ± 3.1	22.6 ± 3.3	21.0 ± 2.7
HC (cm)	98.9 ± 7.2	101.3 ± 6.6	97.0 ± 7.1
WC (cm)	74.0 ± 8.8	80.6 ± 7.5	68.8 ± 5.9
BMR (kJ)	6710.6 ± 1176.9	7820.6 ± 767.4	4757.0 ± 519.1
Fat percentage (%)	19.4 ± 7.0	14.9 ± 5.4	22.9 ± 6.0
FFM (kg)	53.0 ± 10.2	62.9 ± 6.1	45.2 ± 4.2
Water percentage (%)	58.6 ± 5.5	61.5 ± 4.3	56.3 ± 5.2
Muscle percentage [%]	76.6 ± 6.6	80.9 ± 5.1	73.2 ± 5.7
Bone mass (kg)	2.7 ± 0.5	3.1 ± 0.3	2.3 ± 0.2
ECW/ICW	0,7 ± 0.0	0.7 ± 0.0	0.7 ± 0.4
PA (°)	5.9 ± 0.8	6.5 ± 0.6	5.4 ± 0.5

BMI—body mass index; BMR—basal metabolic rate; ECW/ICW—extracellular water/intracellular water; FFM—fat-free mass; HC—hip circumference; PA—phase angle; WC—waist circumference.

**Table 3:** Physical activity of the studied population depending on sex.

Physical Activity (MET, min/week)	Total (Mean ± SD)	Men (Mean ± SD)	Women (Mean ± SD)	Student’s *t*-Test Value	*p* Value
Total MET	13,718.7 ± 1480.2	13,832.1 ± 1688.7	13,629.6 ± 1307.8	0.6	0.560
Sedentary	5368.4 ± 871.9	5166.1 ± 849.1	5527.4 ± 866.3	−1.8	0.075
Light	4244.2 ± 1006.3	4380.1 ± 1128.8	4137.4 ± 898.1	1.0	0.303
Moderate	3554.3 ± 1338.8	3644.9 ± 1328.2	3483.1 ± 1358.9	0.6	0.532
Vigorous	551.8 ± 661.5	640.9 ± 689.7	481.7 ± 638.1	1.3	0.211

MET—metabolic equivalent of work.

**Table 4:** Statistically significant correlation coefficients (*p* < 0.05) between the intensity of physical activity and anthropometrics and body composition characteristics.

Pearson’s Correlation (r)	Physical Activity			
	Amount of Time for Different Levels of Physical Activity			Total MET
	Light	Moderate	Vigorous	
Height	-	-	-	-
Body mass	-	0.494 **	-	-
BMI	-	0.280 * 0.523 **	-	0.357 **
BMR	-	0.536 **	0.411 **	0.345 **
Fat percentage	-	-	−0.272 * −0.418 ***	-
FFM	-	0.528 **	0.423 **	-
Water percentage	-	-	0.232 * 0.335 ***	-
Muscle percentage	-	-	0.273 * 0.419 ***	-
Bone mass	−0.347 **	0.520 **	0.432 **	-
ECW/ICW	0.356 **	-	−0.429 * −0.443 *** −0.385 **	-
PA	-	-	0.303 * 0.346 **	0.235 * 0.338 ***

BMI—body mass index; BMR—basal metabolic rate; FFM—fat-free mass; ECW/ICW—extracellular water/intracellular water; MET—metabolic equivalent of work; PA—phase angle; * total group; ** men; *** women.

**Table 5:** The influence of diverse types of physical activity on chosen anthropometrics and body composition elements in men and in women.

Intensity of Physical Activity	Body Mass * ^a^		BMI * ^b^		BMR * ^c^		Muscle Percentage ** ^d^	
	β	*p* Value	β	*p* Value	β	*p* Value	β	*p* Value
Sedentary	0.369	0.215	0.336	0.254	0.240	0.149	−0.279	0.397
Light	0.265	0.468	0.408	0.263	-	-	−0.169	0.522
Moderate	1.073	0.027	1.175	0.015	0.900	0.003	−0.125	0.771
Vigorous	0.093	0.718.	0.104	0.687	-	-	0.535	0.013
Total MET	−0.579	0.245	−0.685	0.167	−0.319	0.248	−0.199	0.604

^a, b, c, d^—separate regression analyses; BMI—body mass index; BMR—basal metabolic rate; MET—metabolic equivalent of work; β—standardized regression coefficient; * men; ** women.

**Table 6:** The influence of diverse types of physical activity on chosen anthropometrics and body composition elements in the total group.

Intensity of Physical Activity	Fat Percentage ^a^		Muscle Percentage ^b^		Water Percentage ^c^		ECW/ICW ^d^		PA ^e^	
	β	*p* Value	β	*p* Value	β	*p* Value	β	*p* Value	β	*p* Value
Sedentary	0.412	0.074	−0.414	0.072	−0.411	0.074	0.351	0.110	−0.120	0.290
Light	0.233	0.293	−0.233	0.294	−0.180	0.415	0.265	0.213	-	-
Moderate	0.435	0.159	−0.432	0.162	−0.383	0.213	0.211	0.473	-	-
Vigorous	−0.355	0.029	0.354	0.029	0.364	0.024	−0.430	0.006	0.288	0.013
Total MET^5^	−0.062	0.831	0.063	0.832	−0.024	0.935	−0.022	0.939	-	-

^a, b, c, d, e^—separate regression analyses; ECW/ICW—extracellular water/intracellular water; PA—phase angle; β—standardized regression coefficient.

## Data Availability

Study data can be made available upon documented request.
